# “Sometimes, it just stops me from doing anything”: A qualitative exploration of epilepsy management in people with intellectual disabilities and their carers

**DOI:** 10.1016/j.yebeh.2016.09.029

**Published:** 2016-11

**Authors:** Silvana E. Mengoni, Bob Gates, Georgina Parkes, David Wellsted, Garry Barton, Howard Ring, Mary Ellen Khoo, Deela Monji-Patel, Karin Friedli, Asif Zia, Marie-Anne Durand

**Affiliations:** a Centre for Health Services and Clinical Research, Department of Psychology, University of Hertfordshire, Hatfield, UK; b Institute for Practice, Interdisciplinary Research and Enterprise (INSPIRE), University of West London, UK; c Learning Disabilities Services, Hertfordshire Partnership University NHS Foundation Trust, St Albans, UK; d Norwich Medical School and Norwich Clinical Trials Unit, University of East Anglia, UK; e Department of Psychiatry, University of Cambridge, School of Clinical Medicine, Box 189, Cambridge Biomedical Campus, Cambridge CB2 2QQ, UK; f Research and Development, Hertfordshire Partnership University NHS Foundation Trust, St Albans, UK; g NIHR Clinical Research Network: Eastern, Division 4, Mental Health, UK; h Centre for Research in Primary and Community Care, University of Hertfordshire, Hatfield, UK; i The Dartmouth Institute for Health Policy & Clinical Practice, Dartmouth College, Lebanon, NH, USA

**Keywords:** Epilepsy, Intellectual disabilities, Self-management, Qualitative study

## Abstract

**Purpose:**

Epilepsy affects 1 in 5 people with an intellectual disability (ID), but little is known about their experiences of living with epilepsy. A qualitative study was conducted to investigate the impact and management of epilepsy in people with ID.

**Materials and methods:**

People with epilepsy and ID and their carers were invited to take part in semi-structured interviews. Eleven participants with ID and their carers were interviewed together, one participant with ID and their carer were interviewed separately, two interviews took place with the participant with ID only, and one interview took place with the carer only. The interviews were transcribed verbatim, coded, and analyzed thematically (dual independent coding for 30% of the transcripts).

**Results:**

Three themes emerged (participant characteristics, living with epilepsy, epilepsy management and information needs) which indicated the following: 1) diversity regarding health profiles, communication abilities, severity of epilepsy, perceived control of epilepsy, and support needs; 2) a reduction in severity and frequency of seizures for a sizeable proportion of participants through antiepileptic drugs; 3) the lifelong impact of epilepsy and related seizures on participants' activities and quality of life; 4) the perceived burden of epilepsy and difficulty managing the condition for a large proportion of participants; 5) high levels of satisfaction with epilepsy-related services and care; and 6) an overall lack of written accessible information about epilepsy.

**Conclusions:**

This study has highlighted a significant impact of epilepsy and related seizures on the daily lives and quality of life of people with ID. Although a sizeable proportion of participants and their carers considered their epilepsy to be well controlled, the majority reported difficulties managing epilepsy and minimizing its impact on their wellbeing. Excluding care staff and the support provided by epilepsy clinics, the participants had not accessed any adapted self-management or information resources about epilepsy.

## Highlights

• People with ID experience a lifelong impact of epilepsy on their quality of life.
• The effects of seizures can be difficult to manage for people with ID and carers.
• There is a perceived lack of accessible written resources about epilepsy.
• High levels of satisfaction with specialist epilepsy clinics were reported.

## 1 Introduction

People with intellectual disabilities (ID) experience more health inequalities compared with the general population [1], [2]. Epilepsy is a common condition in people with ID, often beginning in childhood and affecting approximately 22% of people with ID compared with 1% of the general population [3], [4]. Seizures are often severe, frequent, and refractory to antiepileptic drugs (AEDs) [5], [6].

In the general population, epilepsy can have a widespread impact on people's lives, including negative effects on psychological wellbeing, adverse AED side effects, disruption to social activities, employment and education, and reduced independence [7], [8], [9]. Although there is less research on the impact of epilepsy on people with ID, poorly controlled epilepsy can severely affect social relationships, education, independence, work, daily activities, and quality of life and can increase mortality and care costs [10], [11], [12], [13], [14], [15], [16], [17], [18], [19]. Furthermore, seizures are a significant cause of preventable hospitalization and premature avoidable death for people with ID [15], [17]. Caring for a family member with ID and epilepsy can also be a significant source of stress and emotional burden for some caregivers, potentially affecting employment, finances, and family activities [20].

In England, the National Institute for Health and Care Excellence (NICE) states that people with ID should be offered the same services, investigations, and therapies for epilepsy as the general population [21]. This includes provision of accessible information and self-management support, which is currently lacking [19]. Self-management and educational interventions in the general population have been found to improve understanding of epilepsy, medication adherence, and seizure frequency [22], [23], [24]. There is little research about epilepsy self-management in people with ID [25], [26], and to the best of our knowledge, the views of people with ID and epilepsy have not been investigated.

Although there are no published, adequately powered and controlled self-management intervention studies for people with epilepsy and ID [25], there is some indication that such support would be useful. A scoping review of self-management interventions for people with epilepsy and ID found five studies completed or underway [26]. One study is a large-scale randomized controlled trial (RCT) that is currently underway [27]. A feasibility RCT has recently been completed with promising findings but was not powered to evaluate efficacy [28]. The three remaining studies did not employ robust designs and methods and included small sample sizes, without appropriate statistical power. However, their findings suggest that tailored interventions may improve the knowledge and self-management skills of people with ID and epilepsy [29], [30], [31].

The present study aimed to explore the impact and management of epilepsy in people with ID. Semi-structured interviews were conducted with people with ID and epilepsy and their carers and sought their perspectives on their lived experiences of epilepsy, the impact on their lives, epilepsy management and care, and the availability and utility of epilepsy information and resources.

## 2 Materials and methods

The WIELD (Wordless Intervention for Epilepsy in Learning Disabilities) study was a randomized controlled feasibility trial exploring key methodological, design, and acceptability issues, in order to subsequently undertake a large-scale RCT to evaluate the effectiveness and cost-effectiveness of an intervention using a picture booklet about epilepsy [32] in adults with ID [28]. The main findings are reported elsewhere [33]. As part of the WIELD study, semi-structured interviews were also carried out with 15 people with ID and epilepsy and/or their carers, which are the focus of this paper.

This study has been reported following the Consolidated Criteria for Reporting Qualitative Studies (COREQ) checklist [34]. As part of the overall WIELD study, the research reported here received ethical approval through the UK National Research Ethics Service.

### 2.1 Participants

Forty participants with ID and epilepsy participated in the WIELD study and were recruited through epilepsy clinics in Hertfordshire Partnership University NHS Foundation Trust in England (all participants were patients of this NHS Trust). Inclusion criteria included diagnoses of ID and epilepsy, at least one seizure within the last 12 months, meaningful verbal or nonverbal communication enabling the participant to engage with the picture booklet intervention, and a carer with sufficient English proficiency to complete the study questionnaires. Participants had a range of communication styles and severity of ID, and the sample included participants with and without capacity to consent to the study. For those without capacity, a carer acted as a consultee.

As part of the overall WIELD study, we invited a subgroup of participants to take part in semi-structured interviews about their experiences of participating in the study (reported elsewhere [33]) and their experiences of living with epilepsy. We aimed to interview 15 participants from the total sample, along with their carers.

### 2.2 Data collection

The WIELD study randomized participants to a control (treatment as usual) or intervention condition and collected questionnaire follow-up data for 20 weeks. At the 20-week follow-up, a subset of participants and carers were telephoned and invited to take part in a semi-structured interview with a researcher. Purposive sampling was used to ensure that a range of views and backgrounds were represented. As the interview schedule was also designed to collect information about the intervention to inform a future full-scale trial, we aimed to interview more participants from the intervention group than from the control group.

Interview schedules were created, including an accessible version with simple questions and images, which were systematically shown to the participant with ID (see supplementary file). The interview schedules were not pilot tested but were developed with the input of researchers, clinicians, people with ID and epilepsy, and their carers.

All participants and carers chose to conduct the interview in the participant's home. All had given consent for audio-recording at the beginning of the WIELD study, except for one carer, whose interview was recorded with hand-written notes. Field notes were not made systematically and therefore were not included in the analysis.

All interviews were conducted by a female postdoctoral psychology research fellow with experience and training in qualitative research and working with people with ID (SM). Prior to the interviews, SM had primarily communicated by phone and letter with carers and had also spoken to some participants. Before the interview began, the purpose of the interview was explained to the participants and carers, and it was stated that SM was a member of the WIELD research team.

### 2.3 Data analysis

The semi-structured interviews were transcribed and analyzed using thematic analysis [35] on the computer software program, NVivo [36]. Two members of the research team coded the interviews (SM and M-AD), with 30% of the interviews being coded by both researchers. Codes were compared and discussed, and themes were derived through discussion.

The sample size was determined prior to analysis. Data saturation was not a factor in determining how many interviews were conducted. Because of time constraints, transcripts were not returned to participants for comments, and the analysis was not discussed with participants.

## 3 Results

### 3.1 Participants and carers

We aimed to interview the participant with ID wherever possible (dependent on the participant's communication abilities) and also requested that a carer involved in the WIELD study was present to seek their views on acceptability and feasibility. The target sample size was fifteen participants, and this was achieved. No participants withdrew from the interview. Two control group participants and two intervention group participants were invited to participate in the interview but declined: two participants declined without providing a reason, one carer was not available, and another carer considered that the interview would increase the participant's anxiety.

Eleven participants with ID and their carers were interviewed together, one participant with ID and their carer were interviewed separately because of being unable to arrange a time to visit them together (participant 42), two interviews took place with the participant with ID only (participants 97 and 98), and one interview took place with the carer only (participant 138). The median length of interviews was 31 min (8–56 min).

The demographics of the participants are shown in Table 1. The interview sample generally reflected the overall WIELD sample, with the exception of more participants being drawn from the intervention group because of the purposive sampling described above.

### 3.2 Themes

The themes that emerged from the interviews following thematic analysis are shown in Table 2: participant characteristics, living with epilepsy, and epilepsy management and information needs.

#### 3.2.1 Theme 1: participant characteristics

##### 3.2.1.1 Additional physical or mental health problems

Participants and carers described the participant's health and healthcare in general and related to epilepsy. Five participants and/or their carers reported that the participant had comorbid health conditions, for which they were seeing professionals and/or taking medication. A range of physical and psychological health issues were discussed, with depression being most frequently mentioned.

*“To get me out of the house for the agoraphobia and all that, my mum took me down to the craft shop once a month to do it but the last, well since my depression flared up again I just, I can't get into any more”*

Participant 97

##### 3.2.1.2 Variation in communication abilities

Participants with ID had mixed levels of communication. Some carers considered their family member or client to have good understanding and expressive skills, whereas other carers and participants noted difficulties in expression and/or understanding.

*“She can't express how she feels, and stuff a lot of the time”*

Family carer of participant 01

For one participant with limited verbal communication, the paid carer highlighted that people who knew them were able to interpret wishes.

##### 3.2.1.3 Satisfaction with their living situation, care, and support

Paid carers and family carers discussed the high levels of support available for participants; for example, always having a staff member accessible and available in a supported living setting. Most participants reported being satisfied with their living situation, care, and support. The majority of participants were not in education or employment, but many participants and their carers spoke of having a full timetable of activities (e.g., attending day centers, taking part in social activities, and volunteering). Seven participants attended a day center, where they interacted with others and engaged in various activities. Four participants or their carer mentioned the participant's active lifestyle and their enjoyment of social activities.

*“Monday and Thursday I go to activity centre. We cook for people, we were making jam yesterday. Wednesday I'm at Health Food Gardens down the road and Friday's my shopping day. Tuesday the Social Worker, she's trying to sort something out. Always seem to be on the go.”*

Participant 118

*“He goes out every other week on a Wednesday and sometimes he goes to the shops and sometimes he'll see a friend of his, and he seems quite happy most of the time”*

Paid carer of participant 42 discussing the participant's activities and social life

##### 3.2.1.4 Varying, but stable, levels of support

For those who did not live with their family, many had been living in their current home for a long period of time. As such, some of the paid carers reported knowing the participant for a long time. Participants received different levels of support from paid carers, and care was often provided flexibly. Some family carers reported providing care around the clock; although the need for one-to-one direct support varied, they had to be available at all times.

*“she lives with us and she can't be left alone, so she can't ever be left unsupervised and she doesn't sleep so and if she does it's small amounts of time”*

Family carer of participant 16

#### 3.2.2 Theme 2: living with epilepsy

##### 3.2.2.1 Long history of epilepsy

Although some participants and paid carers were unable to state when epilepsy was first diagnosed, most reported that epilepsy had been present since birth or childhood. One participant had been diagnosed within the last year and had had blood tests, a MRI, and a CAT scan as part of the diagnostic process. A minority of other participants or carers mentioned diagnostic tests. For five participants, seizures used to be more regular and/or more severe in nature, and they were now perceived to be better controlled. For some participants, seizures had caused injury or required hospitalization, and this created anxiety and distress.

*“My staff do it here for me, that's what I was saying, but otherwise very very bad, back to the doctors and nurses in the hospital again. But apart from that but it's them I haven't had them very very very bad anymore”*

Participant 28 discussing epilepsy management at home compared to when they used to have to go to hospital

*“Do you worry about anything? Mainly start, with the shakes, and the tremors.”*

Participant 51 and paid carer discussing concerns about epilepsy

##### 3.2.2.2 Wide variation in nature of seizures

There was wide variation in the type, frequency, severity, and aftereffects of seizures experienced by the participants. Some carers and participants reported the absence of seizures over the course of the 20-week WIELD study, whereas others reported having several seizures a week up to several seizures a day. Carers were often able to report triggers for seizures that commonly included tiredness, heat, anxiety, stress, illness, hormonal changes, and excitement. Participants were not always able to identify triggers, but where possible, they tried to adjust their lifestyle accordingly.

*“'Cos I try to look after them myself by making sure that I rest, when I'm tired, that's when I'm known to have an epileptic fit, so when I get tired in the day, like today I've had to be on my bed because I've got tired earlier”*

Participant 98

The effect of seizures varied widely, with some people recovering quickly after a seizure, whereas for others, this could take several hours or even several days. Some participants and carers reported falls and injuries during seizures. Fatigue and confusion were commonly reported after a seizure.

*“She's usually very confused, you know, she gets confused after, when she's coming out of it and she does have like rasping, breathing, that kind of thing.”*

Carer of participant 149 discussing the effect of seizures on the participant

One carer of a participant with limited verbal communication felt that the participant probably did not know that she had seizures. A few participants were able to talk about experiencing seizures, and for those who were conscious during seizures, participants and carers reported that it was an unpleasant experience and one that could cause distress. Several participants reported dreading future seizures and their aftermath.

*“Yeah there's lots of drawbacks, lots of drawbacks, and for [participant] obviously not pleasant while she's having them, especially because she's aware, unless she's had the full tonic clonic, and er, and so obviously when she's having them we just talk her through it….mmm just keep trying, and obviously keep saying it's not her fault”*

Family carer of participant 01

*“I know what happens, what happens to me, I go very quiet and I look down at my feet and I won't be able to talk, I can hear you, but I can't respond. And that's when I know it's… when I'm having a fit, and when I've come out of it I'm really tired.”*

Participant 98

#### 3.2.3 Theme 3: epilepsy management and information needs

##### 3.2.3.1 Burden of epilepsy on participants, carers, and family

Dealing with epilepsy was considered a burden by a sizeable proportion of the participants and carers we interviewed, with a direct impact on everyday life and related quality of life. The condition was perceived to significantly limit participants' actions, work prospects, and activities in and outside their home. For participants who experienced multiple seizures a day, the occurrence and aftermath of the seizures could affect the entire day and prevent the participant from engaging in any activities. Some participants craved more independence, which was significantly limited by seizures that were difficult to control.

*“It does get to the stage, where sometimes when she has had more than one in a day, that's it the days, you can't go out, you can't do anything, so it has that impact as well.”*

Carer of participant 01

*“Sometimes, it…it just stops me from doing anything.”*

Participant 51

*“There could be one day where I get really frustrated because of it [epilepsy], and you know, because I can't do the things that I want to do, like I put up this wall about driving but lately I've been saying I want to get, I really do want to get stable so I can drive.”*

Participant 97

Even for participants who reported feeling in control of their condition, seizures were described as extremely unpleasant and tiring, with a recovery time that varied between participants and could last for two days.

*“It's not difficult 'cos I know what to do for it, I don't like having epilepsy because it makes me have seizures and it makes me feel horrible after, I feel groggy, tired, and I can't focus, I can't focus when, when I've had epilepsy I need quietness, I can't stand noise when I come out. And it's horrible.”*

Participant 98

Another contributing factor to the perceived epilepsy burden was participants' anxiety and worry about their condition and associated seizures. Six participants or their carers reported recurrent anxiety about upcoming seizures, potential falls and injuries, hospitalizations, or any epilepsy-related event that would disrupt their routine and activities.

*“I think he worries about having seizures, he thinks that nearly anything can set them off, he thinks, he used to say ‘oh, don't want to work on the computer too long, it'll set off a seizure’, and we had to explain it's doubtful, so yeah, I think he worries about having them.”*

Carer of participant 42

Four participants reported dealing with epilepsy and related seizures well and considered being in control of their condition. They seemed to have developed adequate coping mechanisms and felt empowered to manage epilepsy with their carer(s).

*“I know I can keep more control of them now”*

Participant 68

*“Not nice but there's nothing I can do 'cos I'm coping with it.”*

Participant 112

*“No, you just, with epilepsy yeah it's hard at first, you get stable, you have your ups and downs and get on with it.”*

Participant 97

Several carers reported being confident in dealing with seizures and knew when to alert others and how to react during a seizure.

*“Our family knows exactly how to react, what to do, you know, so that helped us get over it like, you know, get to know what happens and how to handle it when she gets seizures.”*

Family carer of participant 112

Generally, carers had learned to live with the participant's epilepsy and manage seizures adequately, even though seizures were initially anxiety-provoking and difficult to manage. Some reported receiving support from other family members and from professional care staff. One family carer reported experiencing guilt when they had not noticed warning signs of an upcoming seizure or failed to prevent it or limit its impact.

*“I get on with it, it's not use sitting down and cry, it's like you, if you look at it could be somebody that's worse off than what I am, so it's like we've learned to live with it, it's not nice”*

Family carer of participant 112

##### 3.2.3.2 Mixed experiences and needs regarding information and support

Regarding information needs and support, participants and carers reported mixed experiences but, overall, described the absence of epilepsy-related information and support tailored to their needs. Five carers reported having received information (n = 3) or training (n = 2) about epilepsy, in writing or from healthcare professionals. From the participants' perspectives, 12 participants or their carer indicated that they had not received any information about epilepsy specifically adapted to their needs. One participant indicated having received information about epilepsy but could not remember its content. Only two participants recalled having attended information or support groups about epilepsy, and they reported finding it helpful. Some participants and carers reported receiving accessible information about other topics but not about epilepsy. Four participants reported receiving verbal information from their carer or from clinicians.

*“The epilepsy nurses gave me lots of information, but not necessarily easy read nothing like that”*

Family carer of participant 01

*“Have you ever been kind of given any kind of information about epilepsy, not the book, anything before that? Have you ever had any leaflets about epilepsy? Not really, no. No, so no easy read or anything? No.”*

Participant 68 and researcher discussing epilepsy related information

*“Nobody else talked to me about epilepsy, it's you [carer] but I don't know about anybody else.”*

Participant 23

One participant who had been recently diagnosed with epilepsy was struck by the lack of information and support provided by the hospital at the time of diagnosis. No information was shared with the participant or his wife; rather, it was shared with care home staff only.

*“They won't let me, the doctor won't tell me nothing, they keep it away, they don't want to tell me yet, so I know they told the staff.”*

Participant 115

Although most participants had not received information about epilepsy, in nearly half of all the interviews (n = 7), participants and carers indicated that they did not feel the need for information anymore. They felt that they were in control of their epilepsy and had developed, over the years, a fairly good understanding of the condition.

*“well don't need to now, I'm alright, not now I'm back to normal now”*

Participant 23 explaining why they do not need any further information or support

Several participants and carers also highlighted that one significant aspect of living with epilepsy was to explain and help other people to understand this condition. One participant described negative reactions and stereotypes about epilepsy now and in her childhood. Three participants and carers actively tried to inform people about the condition, raise awareness, and/or train others about epilepsy.

*“We actually feed back some of that by, to the Skills and Care course, we do a session for them. Part of their module for new carers to Herts, which we enjoy don't we? And then, yeah, for them, seeing somebody who actually has a bit of epilepsy, and he can relate things that are, that is really meaningful, so it's good really.”*

Carer of participant 68 talking about the training they deliver together

##### 3.2.3.3 Satisfaction with clinical management through epilepsy clinics

As would be expected from the recruitment method, most participants and carers reported being followed up and in regular contact with the epilepsy clinics in the local area by consultant psychiatrists and epilepsy specialist nurses. Some participants with controlled epilepsy had been recently discharged, and three participants were under the care of a neurologist. When seizures occurred, the majority of carers and participants could deal with it at home, although some participants with more severe epilepsy were occasionally taken to the hospital after cluster seizures.

The frequency of epilepsy clinic appointments varied greatly between participants: quarterly, every 6 months, and yearly. Participants and carers were very satisfied with the care and support provided by the staff at the epilepsy clinics. Some participants and carers reported feeling empowered, with perceived increased control of epilepsy.

“*Once we went to the epilepsy clinic, it felt a lot more in control of what we were doing. They're [epilepsy clinic staff] all brilliant, in the fact that you could contact them, and helped us out on so many occasions.”*

Carer of participant 01

One participant reported noticing that doctors at the epilepsy clinic and elsewhere tended to interact more with the carer than with the participant.

*“Because when I go to the doctors and to the epileptic clinic, they're talking to mum more than me because you understand more, don't you mum?”*

Participant 18

##### 3.2.3.4 Medication management

Seven participants or their carers reported that epilepsy was fairly stable and well controlled with AEDs. These participants still sometimes experienced seizures, but carers and participants felt that they were as well controlled as they could expect.

*“Well, with her medication, I mean, I would say because she's seen the epilepsy nurse and she didn't think she needed it up, to up it, I would say that was very well controlled.”*

Carer of participant 149

Several participants or their carers reported having to change medications in order to improve seizure control. Changing medications was considered stressful, occasionally causing worse seizures or side effects. The following side effects were described and considered difficult to manage: tiredness and impairments to mood, cognitive, verbal, and physical skills.

*“what happens is if they increase it just by 1* *ml my fits go through the roof, if they decrease it 1* *ml my fits go through the roof, but because I'm still having fits my neurologist was saying why don't we put you on something else, and my reply was, “why don't you just leave my medication alone?””*

Participant 97

## 4 Discussion

The findings of this study illustrate the significant role that epilepsy plays in the lives of people with ID and the importance of effective epilepsy management. Our group of participants were diverse with differing health profiles, communication preferences, and support needs. However, for most participants, epilepsy was described as a chronic condition that had been present since childhood. Although the frequency and severity of seizures may be reduced through medication, epilepsy can have a long-lasting effect on the everyday lives of people with ID and their carers. Many participants and carers described the epilepsy burden and related impact on their lives, activities, mood, and overall quality of life. Generally, people with ID and their carers expressed satisfaction with the service they received from specialist epilepsy clinics, and many considered their epilepsy to be relatively well controlled by medication despite still experiencing seizures. The majority of participants had never received accessible written information about epilepsy. Some participants expressed that this would no longer be useful to them because of their lived experience of the condition and the support of carers in managing seizures.

Previous research has not explored the impact of epilepsy from the perspective of people with ID and their information and support needs. Understanding lived experiences of epilepsy through in-depth qualitative research enables researchers and clinicians to understand barriers and facilitators to epilepsy management and self-management and, in turn, how lives can be improved [37]. This study has shown that people with epilepsy and ID experience a considerable burden from epilepsy. Although this is perhaps most clearly demonstrated on the impact on everyday activities for those patients who currently have frequent or severe seizures, it is also the case for people with relatively well-controlled epilepsy who continue to experience anxiety because of past seizures. It is also noteworthy that participants with ID and their carers seemed to accept that they would be unlikely to reach total seizure control and that a balance was needed between seizure control and AED side effects [38], [39].

People with epilepsy in the general population also experience negative effects on their lives, such as anxiety, lack of independence, discrimination, and impacts on employment and social relationships [17], [18]. Walker et al. [9] outlined a dyadic model of the impact of epilepsy on people with epilepsy in the general population and their support person. Factors that contributed to negative impacts included uncontrolled seizures and requiring high levels of support in general and with self-management. People with epilepsy and ID are more likely to experience these factors, therefore suggesting that the impact of epilepsy on their quality of life, and their carers', may be higher than in the general population.

Accessible information, and a greater involvement in decision-making for people with ID and epilepsy, is recommended in clinical practice guidelines [19], [21]. In the present study, the majority of people with ID and epilepsy and their carers did not recall receiving accessible information about epilepsy. Several participants reported receiving appropriate support and help from their carers. However, there are potential issues with this reliance on the support of carers, particularly so when relying on paid carers. There may be a high turnover of professional carers and, among them, carers who lack understanding about their client's epilepsy [39]. Further, carers may not always engage the person with ID in discussions about epilepsy and treatment decisions [38]. Therefore, there is a need to support people with ID and epilepsy at times of diagnosis and decision-making with appropriate, accessible information that empowers them to make informed decisions.

### 4.1 Limitations

As part of the interviews, we aimed to gather the views of carers who had been involved in the WIELD study, and we also wanted to include people with a range of ID severity and communication styles who may need support from a carer to take part. Therefore, the majority of the interviews took place with people with epilepsy and ID and their carer (11 out of 15), and it was not possible to analyze the views of these two groups separately. As the views of carers have been reported elsewhere [20], [38], [39], it would also be useful to explore the views of people with ID independently from their carer.

The specialist services available for people with epilepsy and ID in the UK vary according to geographical area [25]. This study focused on patients of one NHS Trust that has well-established epilepsy clinics for people with ID. There were high levels of satisfaction with the epilepsy clinics; many patients and carers felt that the clinicians at the epilepsy clinics had helped them reach an acceptable level of seizure control. However, it may be that experiences of epilepsy management and satisfaction with seizure control would vary in different service settings.

The majority of the people with epilepsy and ID in this study had epilepsy for most of their lives. Health and care guidelines and available resources have changed substantially since these patients received their epilepsy diagnosis, which is a critical time for information and support. One participant had received an epilepsy diagnosis within the last 12 months and reported a lack of communication and information, but further research involving people with ID who have recently been diagnosed with epilepsy is warranted. A wider examination of current practices around diagnosis of epilepsy for people with ID would also contribute to our understanding about how best to support people with ID and epilepsy.

### 4.2 Conclusion

Despite one in five people with ID experiencing epilepsy, research on the management and impact of this condition is limited. This study has shown that the majority of people with ID we interviewed have received minimal information about epilepsy, despite still experiencing seizures and effects on their everyday lives. Management of epilepsy is primarily achieved through medication, although in addition to this, some people with ID and their carers reported adapting their lifestyle accordingly. Accessible and targeted information and support have the potential to empower people with ID to play a more active role in their epilepsy management and reduce the impact on their quality of life. We anticipate that tailored information and support would achieve maximum impact if delivered soon after receiving a diagnosis of epilepsy or at times of clinical decision-making, which was not the case in our sample.

## Contributors

M-AD planned and designed the qualitative component of the study. M-AD developed all interview guides, accessible interview guides, and accessible recruitment materials. M-AD applied for the initial NHS ethics application. MEK, DM-P, SM, GP, and AZ recruited participants. SM collected data. SM and M-AD analyzed data. SM and M-AD drafted the article. All authors commented on subsequent drafts and approved the manuscript for publication.

## Ethics approval

This study was approved by Wales National Research Ethics Service (NRES) Committee 5 (Ref: 14/WA/0135).

## Source of funding

This manuscript presents independent research funded by the NIHR under its Research for Patient Benefit (RfPB) Programme (Grant Reference Number: PB-PG-0213-30042).

## Disclaimer

The views expressed are those of the authors and not necessarily those of the NHS, the NIHR, or the Department of Health.

## Trial registration

This trial was registered with the National Institute for Health Research (NIHR) Clinical Research Network Portfolio Network (ISRCTN: 80067039).

## Figures and Tables

**Table 1:** Demographics of participants with ID interviewed for this study.

	Demographics
Gender	
Female:male	9:6
Age	
Mean (standard deviation)	42.60 (15.26)
Ethnicity	
White	14
Black — other	1
Years since diagnosis^a^	
Mean (standard deviation)	31.46 (18.68)
Accommodation	
Family home	4
Own property	1
Registered care home	3
Supported accommodation	6
Tenancy	1
Carer relationship to participant	
Family member:paid carer	6:9
Number of hours of care per week provided by carer	
Median (IQR)	20 (8–168)
WIELD group allocation	
Control:intervention	4:11

a Missing data n = 2.

**Table 2:** Key themes from the interviews with participants and carers.

Themes	Subthemes
Participant characteristics	• Additional physical or mental health problems. • Variation in communication abilities. • Satisfaction with their living situation, care, and support. • Varying, but stable, levels of support.
Living with epilepsy	• Long history of epilepsy. • Wide variation in nature of seizures.
Epilepsy management and information needs	• Burden of epilepsy on participants, carers and family. • Mixed experiences and needs regarding information and support. • Satisfaction with clinical management through epilepsy clinics • Medication management

## Appendix A Supplementary data

Supplementary material. Accessible information sheet for people with ID and epilepsy. Image 1
