# Supplementary material for: “Sometimes, it just stops me from doing anything”: A qualitative exploration of epilepsy management in people with intellectual disabilities and their carers
Source: Epilepsy Behav. 2016 Nov;64(Pt A):133–9. doi: 10.1016/j.yebeh.2016.09.029 (PMC5140003; doi:10.1016/j.yebeh.2016.09.029)
Supplement: Supplementary file 1 — Supplementary material. Accessible information sheet for people with ID and epilepsy. [file mmc1.pdf]

Interview Schedule

<u>What you think of taking part in the WIELD study</u>

The interviewer will:

- Introduce herself.

- Explain the aim and content of the interview, duration, use of audio-recording, and remind the carer and participant that they have already consented to this interview in the initial consent form. However, she will reiterate that the carer and participant are free to stop at any time, without giving any reasons and that this will not affect the standard of care the participant receive in any way.

- Assess the capacity of the person with learning disabilities to take part in the interview, and work with the carer to ensure that the participant understands the questions. If the participant is not able to understand some of the questions and provide answers, the carer can reply on his/her behalf, and use what the carer knows about his/her feelings about the study and experience of taking part in the study over the past 20 weeks in order to answer the questions.

- The Methods of eliciting answers will vary considerably between respondents, reflecting their abilities and needs.

- The images will be presented one by one on a document without any words, as the interviewer asks each question.

- Only the questions written in blue will be used with the person with learning disabilities.

University of West London Connected. University of Hertfordshire UH University of Cambridge UEA University of East Anglia

Interview questions

WIELD study

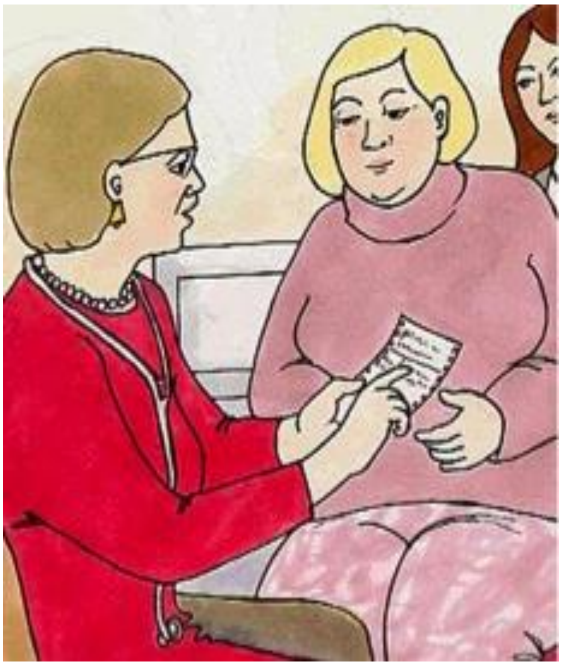

© Sheila Hollins www.booksbeyondwords.co.uk

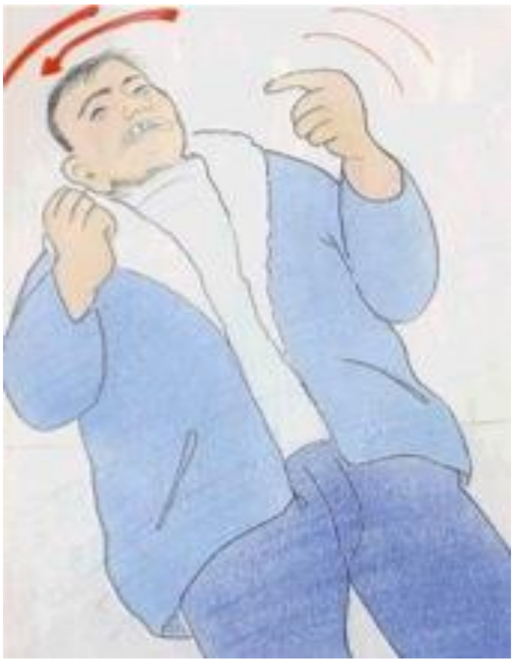

© Sheila Hollins www.booksbeyondwords.co.uk

Can you tell me about you and about your epilepsy?

Probes:

- How often do you come to the Epilepsy Clinic?
- How long have you had epilepsy for/how many years?
- Do you come alone or does someone always come with you?

For the carer:

- What is your relationship to the person with learning disabilities?
- How long have you been caring for [name of the person]?

- o How often do you see [name of the person]? How much time do you spend with him / her?
- o Does he/she live independently?
- o When was has he/she diagnosed with epilepsy? (if previous question not answered)

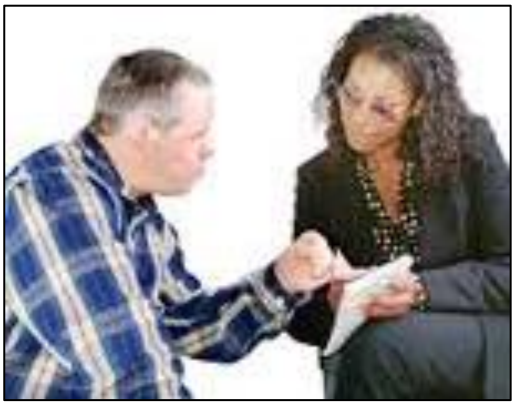

Over the past year and before the study, have you been given information about epilepsy?

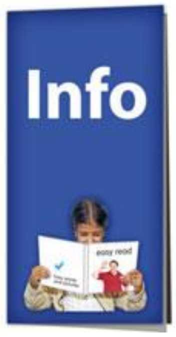

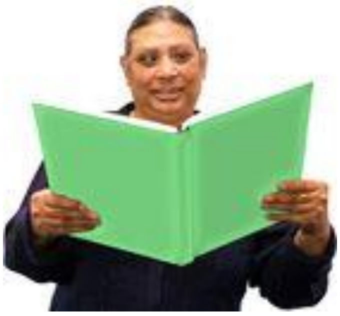

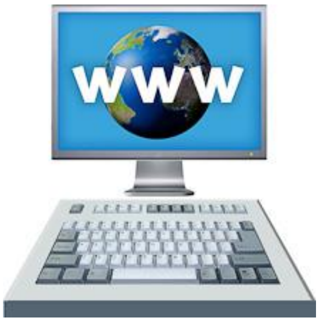

Like a leaflet, a book, or a website?

Probes:

- What was it?
- Was it good/helpful? Did you like it? What could be better?
- Was it easy to understand and to use?
- Where did you find it/who gave it to you?
- When/how often did you use it?
- If you didn't understand something, who did you ask for help?

To discuss with the carer if necessary:

- o What were they, and what did you think of those resources?
- o How did you find out about those resources?
- o How often did he/she use it?
- o Was it adapted to the needs of people with learning disabilities?
- o Was it easy to use/helpful?
- o What was your role in their use?

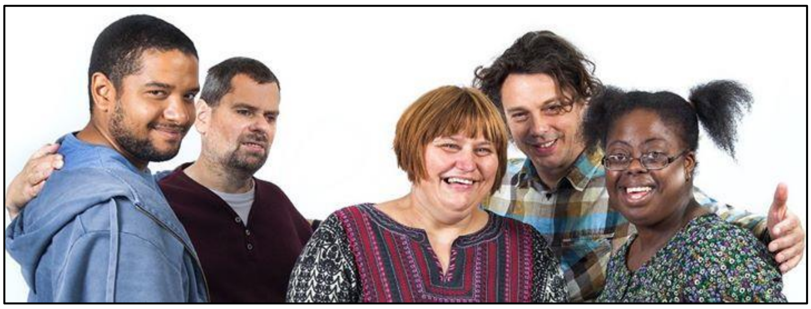

Over the past year and before the study, have you spoken to people or gone to places where people have helped you with your epilepsy?

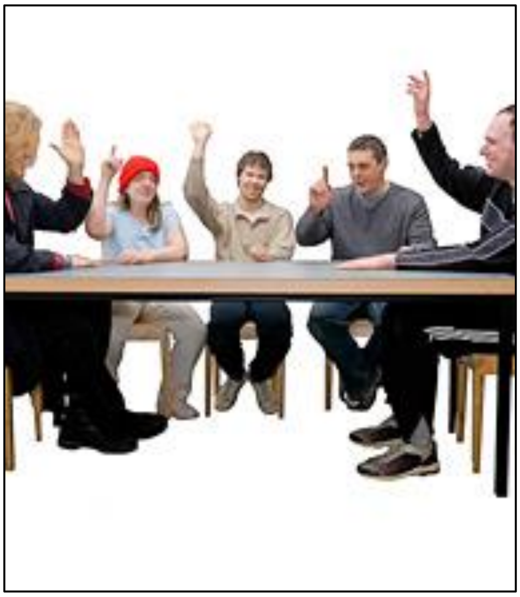

Like going to a group where other people have epilepsy?

Probes:

- What was it?
- Was it good/helpful? Did you like it? What could be better?
- Where did you find it/who gave it to you?
- When/how often did you use it?

To discuss with the carer if necessary:

- What were they and what did you think about these services?
- How did you find out about those services?
- How often did he/she use those services?
- Was it adapted to the needs of people with learning disabilities?
- Was it easy to use/helpful?
- What was your role in the use of these services?

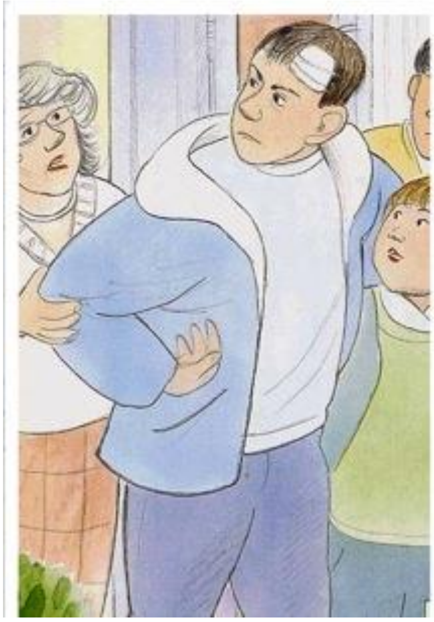

© Sheila Hollins
www.booksbeyondwords.co.uk

What is it like to live with epilepsy?

Probes:

- Is it easy? Is it difficult? What is difficult?
- Do you have enough information?
- What could help you?

To discuss with the carer if necessary:

- Do you feel that he/she has all the information and support that he/she needs?
- As his/her carer, how do you find it?
- Do you think that you would benefit from additional information or support about epilepsy and its management?

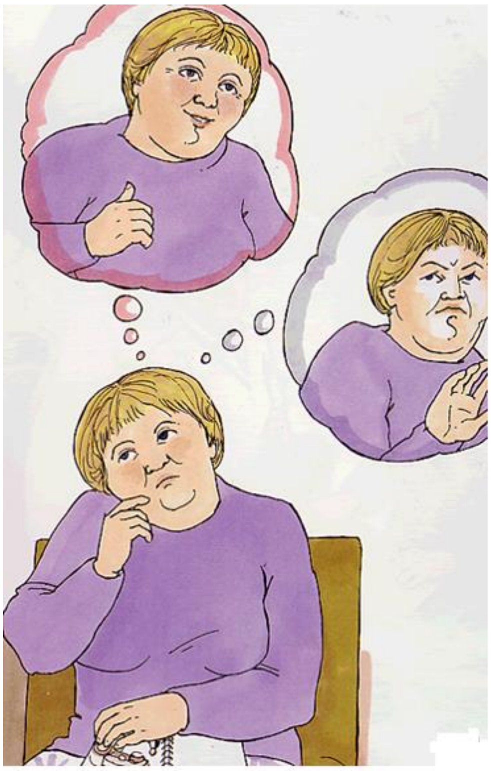

© Sheila Hollins
www.booksbeyondwords.co.uk

What did you think about being in the study?

Probes:

- Was it easy? Was it difficult?
- What did you think about the information leaflet and consent form?
- Was it easy to read?
- Was it too long?
- Was there something missing?
- What could be better?
- What did you think about speaking to the nurse?
- What did you think about speaking to the researcher?

To discuss with the carer if necessary:

- What did you think of the information sheet and consent forms developed for the carer?
- What did you think of the easy read information sheet and consent forms?
- Was it clear and easy to understand?
- Was it too long?
- Was there anything missing?
- What did you think of the initial meeting with the research nurse?
- Could anything be improved about the process of being informed and consented into the study?
- What did you think of your discussions with the Research Nurse and the Researcher?
- What did you think about being randomly allocated to receiving or not receiving the booklet?

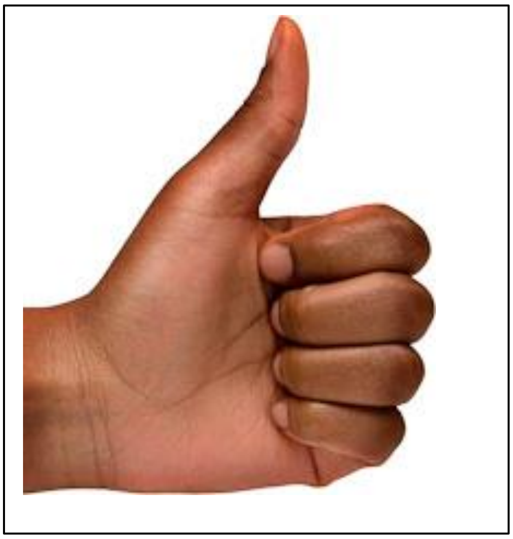

What did you think was good about this study?

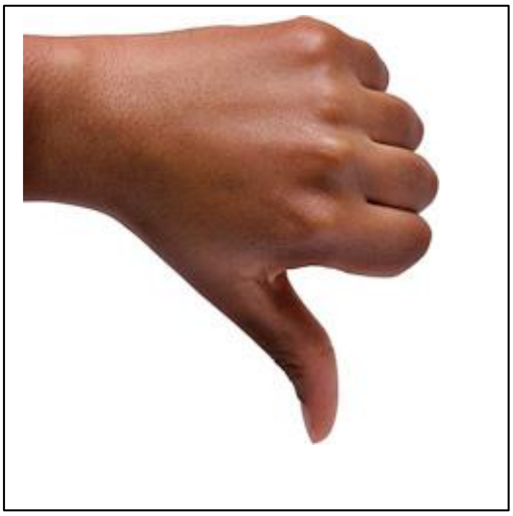

What did you think was bad about this study?

To discuss with the carer if necessary:

- Did it take too much time to be in the study?
- Did you ever feel forced into taking part in the study?

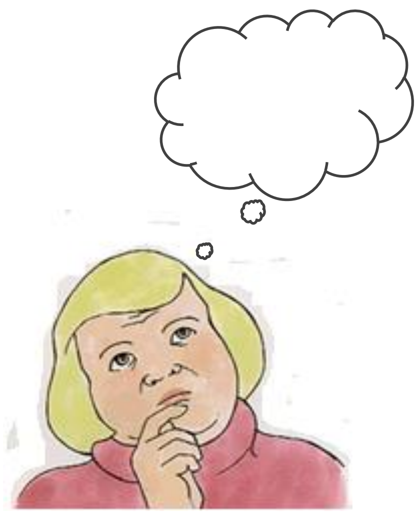

© Sheila Hollins

What do you think could be better?

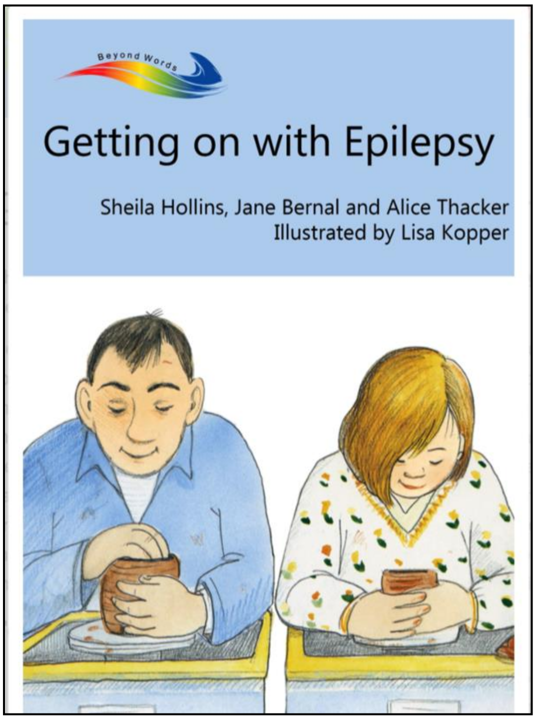

What did you think about the book?

Probes:

- o What was it like to use it with Research Nurse?
- o What was it like to use it at home?
- o What was good?
- o What was not good?
- o What else do you want to say about the book?

To discuss with the carer if necessary:

- o The booklet itself?
- o The black and white images at the end?
- o Using the booklet with the Research Nurse?
- o Using the booklet at home?
- o What did you like about the booklet?
- o Was there anything about the booklet that you did not like?
- o Online video and training slides provided by Books Beyond Words?

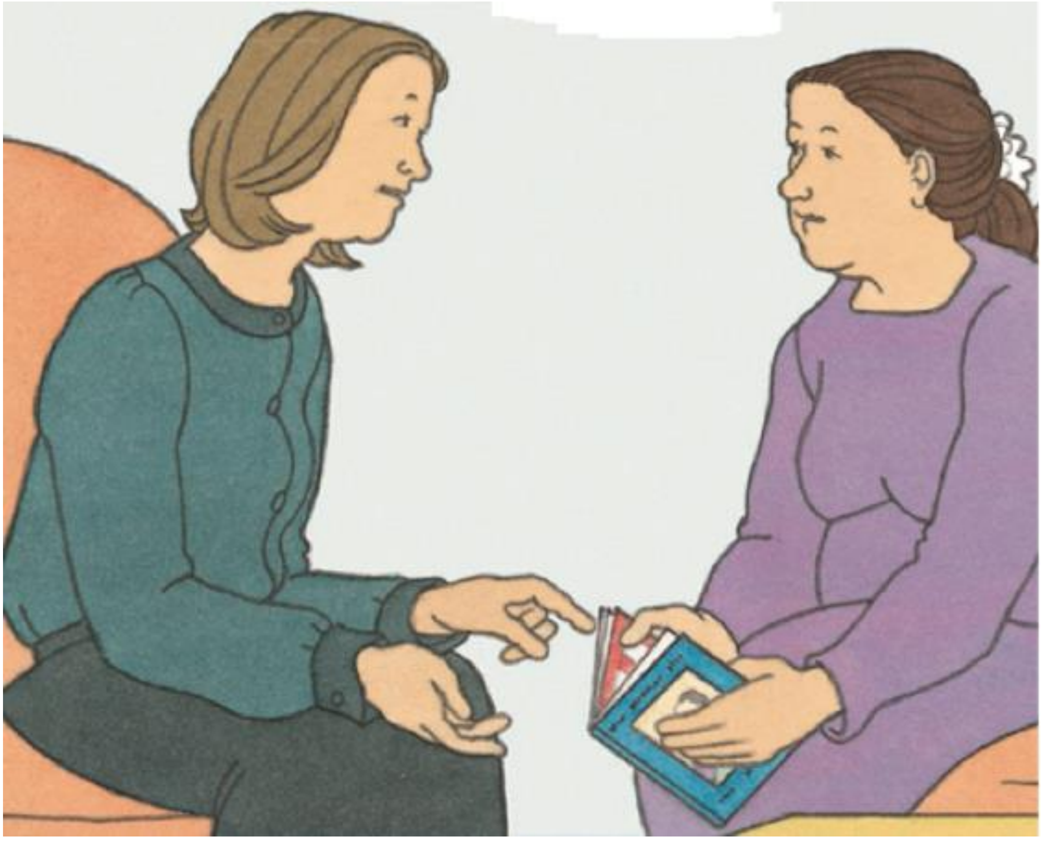

© Sheila Hollins www.bookbeyondwords.co.uk

Would it be good to use this book with someone who has learning disabilities and epilepsy?

Probes:

- o Would other people with learning disabilities like to use it?
- o Would it be helpful for them?

To discuss with the carer if necessary:

- o Would it be easy enough for carers and people with learning disabilities to use without a lot of external support?
- o Would it be helpful/worth it?
- o Would it be too time-consuming?

- o How do you think other people with learning disabilities would react if they were given this book?
- o How do you think other carers would react if they were given this book?
- o How do you think health professionals would react?

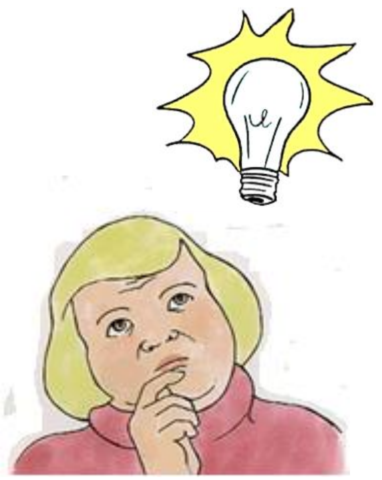

© Sheila Hollins

Do you want to say something else about being in the study?

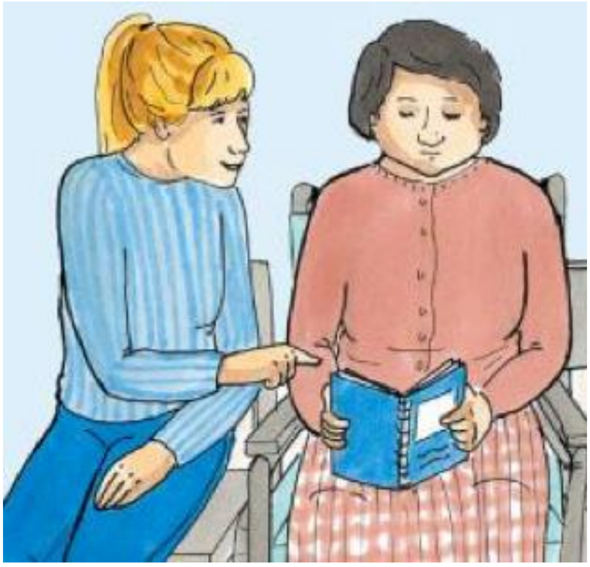

© Sheila Hollins www.bookbeyondwords.co.uk

Would you tell other people with learning disabilities to be in the study

For the carer only:
I would like to talk to you specifically about the questionnaires.

How did you find it to complete the questionnaires?

Probes:

- Did you find it easy/difficult?
- How did you feel about completing the questionnaires four times?

Do you think they can be improved in any way?

Probes:

- Relevance;
- Language used;
- Time taken;
- Design;
- Posting the questionnaires.

Do you have any other comments about your participation in this study?

Or any other comments about the study in general?

Would you recommend participating in a similar study to other people with learning disabilities and carers?

Probes:

- Could you please explain why?

Debriefing

Thank the patient and carer for participation in the interview, answer any questions they might have and ensure they understand what will happen with the findings.
